# Event and Comment

**Published:** 1910-04-15

**Authors:** 


### DENTAL REGISTER.

Vol. LXIV. April 15, 1910. No. 4.

### EVENT AND COMMENT.

DENTAL ANESTHETICS—From the oldest records of dental operations, the pain incident thereto has been a most important source of complaint on the part of the patient. The early dentists tried by every known art to minimize this terrifying accompaniment, by hypnotism or suggestion, and by technical procedure which would cause the teeth, for instance, to loosen from their attachments and be easily removed with the fingers, and other similar unoffensive means. In the fourteenth century. Guy de Chauliac, according to Guerini's History of early Dentistry writes of a method of administering anesthetics that might have been written in modern times. He says: "Some prescribe medicaments which send the patient to sleep, so that incisions may not be felt: such as opium, the juice of the morel, hyoscyamus, mandrake, ivy, hemlock, lettuce, etc., A new sponge is soaked in juices of these drugs and left to dry in the sun: and when there is need for it the sponge is dipped in warm water and held under the nostrils of the patient until he goes to sleep. Then the operation is made." To arouse the patient from the anesthesia, another sponge is soaked in vinegar and held under the patient's nose. All of which shows that our modern methods of anesthesia are not so new as they are claimed to be. There can however, be little question as to the value and increasing use of various methods of eliminating the pain incident to dental operations. Our technic and materials have greatly increased in modern times, so much so that no one now attempts operations involving much pain without using some form of anesthetic. In spite of the fact that we now have so many approved anesthetics and our knowledge of their powers and methods of administration have become so well known and practicable, we still find many dentists inflicting serious pain on patients with no special effort to avoid it. There are several reasons for this, some excusable, but mostly they are wholly injustifiable. There are few dental operations that can not be made comfortably painless, providing the operation has the technical knowledge and skill required and is willing to spend the time required. There can be no justifiable reason why any one who practices dentistry to-day should not have the knowledge required, as we have many excellent text books and much current literature on the subject, and it is not impossible for even the busiest practitioner to make time enough to read this literature and inform himself. The question of attaining the necessary skill is a more difficult question, and yet not beyond the capacity of the practitioner of average ability in other technical lines. A knowledge of the best methods and perseverence in well directed efforts to master them will bring big rewards to any one who takes up the work earnestly. If we could only believe there was an anesthetic possibility for every distressing pain, and that we are charged with an obligation to administer this relief in every case, this should be incentive enough to stimulate any one to do his best to master the art. The objection that we most frequently hear made is that all anesthetics are poisonous or detrimental to the health of our patient or the tissues on which we work. This is merely an excuse for laziness, as every one can know the dangers and how to avoid them, and at the worst there are probably many times more frequent and serious results from shock without anesthetics than fatal results from their use. Anesthetics are dangerous but this is all the more reason why we should study them most carefully and know the peculiarities of their reactions on the system and the tissues to which we apply them. Many dentists are making large use of these agents to the immense comfort and advantage of their patients and because they have studied to perfect their knowledge they do not fear their powers but utilize them to their full capacity. There is also another and quite important reason why more use is not made of anesthetics, both general or systemic, and the local obtundent remedies, and it is the time consumed in their application and the necessary confusion sometimes introduced into the well ordered discipline of an office practice. Many times the obtunding of sensitive dentine for painless excavation because of peculiar conditions of the tooth structure, location of the cavity, and the like, will necessitate delay or more or less experimenting or changing of anesthetics or apparatus. Much valuable time is in this way consumed, interrupting or delaying the order and discipline of the office, much to the discomfort of patients and operator. But after all it may be well to encounter criticism and suffer annoyance from unexpected delays rather than inflict serious pain and cause results that forever condemn our practice as one to be shunned at all hazards. Do we not owe a duty to ourselves as well as to our patients in this matter? We cannot but feel that our profession has not considered this very important unction of practice as at all obligatory from any standpoint except where it was absolutely demanded or where it ministered mostly to a monetary advantage. We become so accustomed to inflicting slight pains without serious protests that we habitually fail to discriminate, and many times overstep the bounds of reason and judgment, which should be matured by the most careful and constant study of conditions requiring such interfeience. We are printing in this issue a symposium of papers setting forth this subject in its various phases which we believe everyone will read with profit.

ORAL HYGIENE CAMPAIGN—At Cleveland, March 18th, was held one of the most interesting conventions we have ever attended. The Oral Hygiene Committees of the National, Ohio State and Cleveland Dental Societies conducted a meeting of two sessions which was attended by upwards of two thousands dentists, school teachers and others interested in the propaganda of caring for the teeth of school children in some practical manner. Six clinics have been installed in as many of Cleveland public schools which were inspected by the visitors. At the general meetings addresses were made by the superintendent of Instruction of Cleveland, physicians and dentists and others interested in similar lines of work in other callings. There is no doubt but the people are getting awakened to the value and need of this work, and a great problem is about to be placed before the dental profession for solution. Just how we are going to meet it and solve it, is one of the important and first questions we shall have to answer.

If this meeting, with its great enthusiasm, is an indication of a general awakening all over the country, we are confident the profession is wholly incompetent at present to give it anything like adequate attention, our colleges must get busy and furnish us a large quota of recruits at once.

